# Expression of *Tmem119*/*Sall1* and *Ccr2*/*CD69* in FACS-Sorted Microglia- and Monocyte/Macrophage-Enriched Cell Populations After Intracerebral Hemorrhage

**DOI:** 10.3389/fncel.2018.00520

**Published:** 2019-01-09

**Authors:** Qian Li, Xi Lan, Xiaoning Han, Jian Wang

**Affiliations:** Department of Anesthesiology and Critical Care Medicine, The Johns Hopkins University School of Medicine, Baltimore, MD, United States

**Keywords:** fluorescent-activated cell sorting, intracerebral hemorrhage, monocyte-derived macrophage, magnetic-activated cell separation, microglia

## Abstract

Activation and polarization of microglia and macrophages are critical events in neuroinflammation and hematoma resolution after intracerebral hemorrhage (ICH). However, distinguishing microglia and monocyte-derived macrophages histologically can be difficult. Although they share most cell surface markers, evidence indicates that the gene regulation and function of these two cell types might be different. Flow cytometry is the gold standard for discriminating between the two cell populations, but it is rarely used in the ICH research field. We developed a flow cytometry protocol to identify and sort microglia and monocyte-derived macrophages from mice that have undergone well-established ICH models induced by collagenase or blood injection. In addition, we combined a recently established magnetic-activated cell separation system that allows eight tissue samples to be assessed together. This protocol can be completed within 5–8 h. Sorted cells are fully preserved and maintain expression of microglia-specific (*Tmem119/Sall1*) and macrophage-specific (*Ccr2/CD69*) markers. They retain phagocytic ability, respond to lipopolysaccharide stimulation, and engulf fluorescent latex beads. Thus, this protocol represents a very important tool for researching microglial and monocyte-derived macrophage biologic function after ICH and other brain diseases.

## Introduction

Intracerebral hemorrhage (ICH), the second most common type of stroke, affects more than 1 million people each year (Kim and Bae, 2017; Thabet et al., 2017). As the most important innate immune cell types, microglia and infiltrating monocyte-derived macrophages play critical roles in neuroinflammation (Wang and Tsirka, 2005; Wang, 2010; Wu et al., 2010; Lan et al., 2017a), hematoma resolution (Flores et al., 2016; Chang et al., 2017; Lan et al., 2018), white matter injury (Li et al., 2017d, 2018), and neuronal toxicity (Wu et al., 2011; Yang J. et al., 2016; Zhang et al., 2017) after ICH. Recent studies have indicated that microglia and macrophages may have opposing functions after ICH: macrophage depletion exacerbates brain damage after experimental ICH (Min et al., 2016), whereas elimination of microglia improves ICH outcomes in the same animal model (Li et al., 2017a). Therefore, distinguishing microglial and macrophage function, activation, and polarization is critical to understanding ICH pathophysiology (Lan et al., 2017c, 2018).

Magnetic-activated cell separation (MACS) has been used to isolate microglia and monocyte-derived macrophages (MMϕ) in mouse models of Alzheimer’s disease (Kronenberg et al., 2017), glioma (Szulzewsky et al., 2015), ischemic stroke (Yang B. et al., 2016), and ICH (Lan et al., 2017a). However, to our knowledge, no protocol exists for separating the two cell types after ICH. Here, we used a MACS tissue dissociation system to dissociate the brains of mice after collagenase-induced or blood-induced ICH. Then, after removing myelin and red blood cells (RBCs), we counted live cells and used fluorescence-activated cell sorting (FACS).

Flow cytometry can provide highly sensitive detection and accurate analysis of different cell populations. In our optimized procedure, we used fluorescent antibodies Ly6g, CD11b, and CD45 together with propidium iodide (PI) to stain brain cells, and subsequently sorted the cells by flow cytometry. The populations of Ly6g^-^/CD45^high^/CD11b^+^ and Ly6g^-^/CD45^Int^/CD11b^+^, which encompass infiltrating monocytes/macrophages and resident microglia, were sorted separately (Hellstrom Erkenstam et al., 2016; Varvel et al., 2016; Vinet et al., 2016). We confirmed that the expression of *Tmem119*/*Sall1* and *Ccr2*/*CD69* differed in FACS-sorted microglia- and monocyte/macrophage-enriched cell populations, respectively. These isolated cells had maximally preserved biologic characteristics after ICH, including inflammatory responses, phagocytosis, and dynamic polarization. They also can be applied to real-time PCR, RNA nanostring, mass spectrometry/proteomics, and *in vitro* cell culture.

This MACS and FACS-based method allows us to distinguish microglia and infiltrating monocytes/macrophages after ICH using MMϕ cell surface markers. This method is fast, efficient, simple, and accurate. Therefore, our optimized protocol provides an important tool for studying MMϕ function after ICH and other brain diseases.

## Materials and Equipment

### Animals

All animal experiments were conducted in accordance with guidelines from the National Institutes of Health and were approved by the Institutional Animal Care and Use Committee at The Johns Hopkins University School of Medicine. Adult male C57BL/6 mice (8–10 weeks old) were purchased from Charles River Laboratories (Frederick, MD).

### ICH Mouse Models

• Collagenase VII-S, cat #C2399, Sigma-Aldrich
• 50-μL Hamilton syringe, cat #80100
• 1-μL Hamilton syringe, cat #80908
• Motorized microinjector,
• DC Temperature Controller 40-90-8D, FHC Inc., ME

### Tissue Dissociation

• Neural Tissue Dissociation kit (P), cat #130-092-628, Miltenyi Biotec
• C Tubes, cat #130-096-334, Miltenyi Biotec
• gentleMACS Dissociator, cat #130-093-235, Miltenyi Biotec
• MACSmix Tube Rotator, cat #130-090-753, Miltenyi Biotec
• Myelin Removal Beads, cat #130-096-731, Miltenyi Biotec
• Myelin removal buffer: PBS solution containing 0.5% bovine serum albumin (BSA)
• Red Blood Cell Lysis Solution, cat #130-094-183, Miltenyi Biotec
• LS columns, cat #130-042-401, Miltenyi Biotec
• QuadroMACS Separator, cat #130-091-051, Miltenyi Biotec
• HBSS with Ca^2+^/Mg^2+^, cat #14025134, Thermo Fisher Scientific
• HBSS without Ca^2+^/Mg^2+^, cat #14170161, Thermo Fisher Scientific
• 70-micron cell strainer, cat #352350, Corning Inc.

### Flow Cytometry and Fluorescence-Activated Cell Sorting (FACS)

• FITC-CD11b, cat #130-081-201, Miltenyi Biotec
• PE-CD45, cat #130-102-596, Miltenyi Biotec
• APC-Ly6g, cat #560599, BD Pharmingen
• BV421-CD45, cat #103133, Biolegend
• Flow buffer (HBSS without Ca^2+^/Mg^2+^, 10 mM HEPES, 1% BSA)
• Blocking buffer (1% goat serum, 0.5% BSA, and 2 mM EDTA in PBS)
• MoFlo cytometer, Beckman Coulter

### Real-Time PCR and Cell Culture

• TRIzol reagent, cat #15596018, Thermo Fisher Scientific
• NanoDrop 2000 spectrophotometer, Thermo Fisher Scientific
• SuperScript VILO cDNA Synthesis kit, cat #11754250, Thermo Fisher Scientific
• TaqMan Universal Master Mix II, cat #4440038, Thermo Fisher Scientific
• Real-time PCR primers, TaqMan^®^Gene Expression Assay, Thermo Fisher Scientific
• QuantStudio^TM^ 3 Real-Time PCR System, 96-well, 0.1 mL
• DMEM/F-12, cat #11330057, Thermo Fisher Scientific
• Fetal bovine serum (FBS), cat #10438026, Thermo Fisher Scientific
• Penicillin-streptomycin, cat #15140148, Thermo Fisher Scientific
• M-CSF, cat #315-02, PeproTech
• Culture medium: DMEM/F-12 with 10% FBS, 100 U/mL penicillin-streptomycin and 20 ng/μL M-CSF
• pHrodo Red Zymosan Bioparticles Conjugate for Phagocytosis, cat #P35364, Thermo Fisher Scientific

### Step-By-Step Procedure

#### ICH Mouse Models: 20 min to 50 min/Each Mouse

**FIGURE 1:**
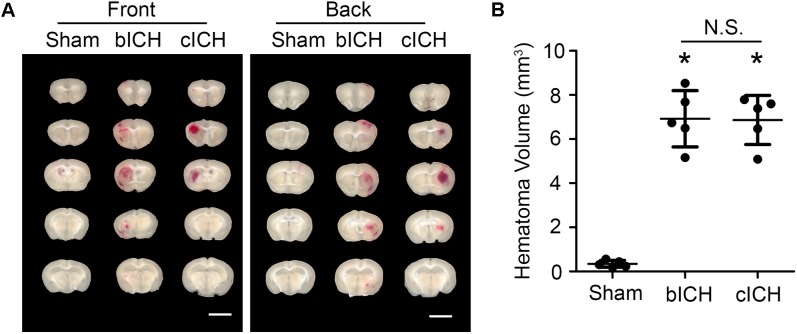
Hematomas on day 1 after blood-induced intracerebral hemorrhage (bICH) and collagenase-induced ICH (cICH) were matched for size. Eight- to ten-week-old male C57BL/6 mice underwent collagenase injection, blood injection, or sham procedure. Mice were sacrificed at day 1 post-ICH. **(A)** Representative images from fresh brain coronal sections. **(B)** Quantification of hematoma volume. ^∗^*p* < 0.05 vs. Sham; N.S., not significant; *n* = 5. Results are presented as scatter plots (mean ± SD). One-way ANOVA followed by Dunn’s multiple comparison post-test. Scale bars: 1 mm.

Mice were anesthetized with 1–3% isoflurane and ventilated with oxygen-enriched air (20%:80%) via a nose cone. We used two well-established ICH mouse models – the collagenase-induced model and the blood-induced model – for this protocol (Li and Wang, 2017). For the collagenase-induced ICH model, we injected collagenase VII-S (0.0525 U in 0.35 μL sterile saline) into the striatum (0.1 μL/min) at the following coordinates relative to the bregma: 0.8 mm anterior, 2 mm lateral, and 2.8 mm deep (Li et al., 2017b; Yang et al., 2017; Zhu et al., 2018). For the blood-induced ICH model, we injected 20 μL of autologous whole blood at a rate of 1 μL/min at those the same coordinates (Zhu et al., 2014; Meng et al., 2017; Wu et al., 2017). We chose the injection volumes based on preliminary experiments in which we matched hematoma volume in the two models on day 1 post-ICH, when hematoma reaches its maximum (Wang et al., 2015), to ensure a fair comparison. Our results showed that the hematoma size induced by 0.0525 U collagenase (6.86 ± 1.11 mm^3^, *n* = 5) was similar to that induced by 20 μL blood injection (6.92 ± 1.27 mm^3^, *n* = 5) at 1 day post-ICH (Figure 1). Therefore, we used those dosages for our subsequent experiments.

Animal core body temperature was maintained at 37.0 ± 0.5°C throughout the surgery and recovery periods with a DC Temperature Controller 40-90-8D. Sham control mice underwent the same procedure, including needle insertion, but without collagenase or whole blood injection.

NOTE: Completion of the procedure requires 20 min per mouse for the collagenase-induced ICH model and 50 min per mouse for the blood-induced ICH model.

#### Brain Tissue Preparation: 10 min/Each Mouse

At 24 h after ICH induction, mice were sacrificed and perfused with ice-cold phosphate-buffered saline (PBS; Thermo Fisher Scientific, Waltham, MA). Based on our pre-calculated injury volume, we collected a 4 mm-thick sample from the ipsilateral caudate putamen (∼80 mg/sample) that included all of the injured area (Chang et al., 2015). Each sample was quickly transferred to 1 mL of cold Hanks’ buffered salt solution (HBSS; without Ca^2+^/Mg^2+^) for the dissociation steps.

NOTE: Sacrifice the mice as soon as possible to keep brain tissue fresh and maximally maintain the live cell status. Delay may result in fewer live cells.

#### Tissue Dissociation With the gentleMACS^TM^ Dissociator: 40 min/up to 8 Mice

**FIGURE 2:**
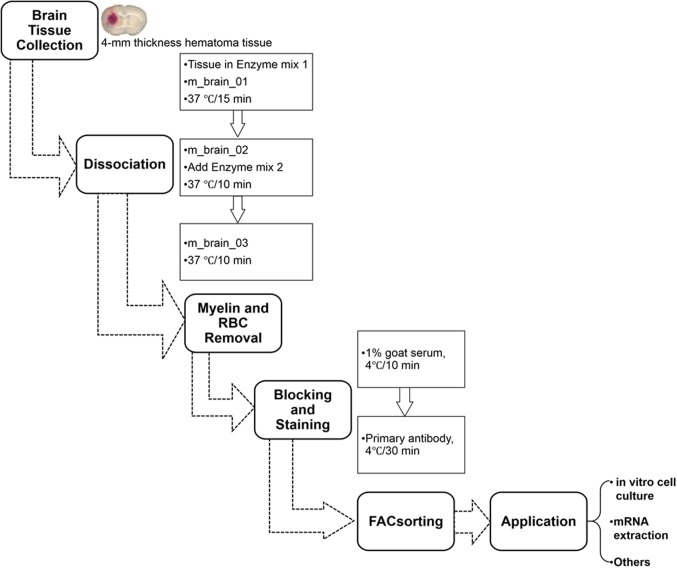
Procedure for isolating mouse microglia and monocyte-derived macrophages after ICH. RBC, red blood cells.

The automated dissociation system (Lee and Lufkin, 2012) was purchased from Miltenyi Biotec (Auburn, CA). The manufacturer’s instructions were followed for reagent preparation and major steps (Figure 2).

(1) Transfer tissues into the C tubes containing 37°C pre-warmed enzyme mix 1 (50 μL Enzyme P and 1900 μL Buffer X).
(2) Run the gentleMACS program “m_brain_01” when C-tubes are attached onto the gentleMACS Dissociator.
(3) Incubate the samples for 15 min at 37°C, and then run on program “m_brain_02.”
(4) Add 30 μL of enzyme mix 2 (20 μL Buffer Y and 10 μL Enzyme A) into each sample and re-incubate them for 10 min at 37°C.
(5) Run on program “m_brain_03” with another incubation for 10 min at 37°C; then spin down quickly (4000 rpm for 10–15 s at room temperature) and collect the cells.
(6) Resuspend the cells gently in 10 mL of HBSS (with Ca^2+^/Mg^2+^) and pass them through a 70-micron cell strainer to a new 50 mL tube.
(7) After washing the cells in 10 mL HBSS (with Ca^2+^/Mg^2+^), centrifuge them at 300 × *g* for 10 min at 4°C and then collect them for myelin and RBC removal.

NOTE:

(a) The gentleMACS^TM^ Dissociator can hold up to eight C tubes at once. Thus, more samples may take more than 40 min.
(b) Before attaching the C tubes to the dissociator, make sure they have been tightened.

#### Sample Clearing: 80 min

All procedures were carried out according to the manufacture’s protocol.

Myelin removal:

• Incubate cells with Myelin Removal Beads (200 μL beads and 1800 μL buffer per sample) for 15 min at 4°C.
• Wash cells in 5 mL of myelin removal buffer and centrifuge at 300 ×*g* for 10 min at 4°C.
• Suspend pellets in 1 mL buffer and apply them onto the LS column (3 columns for each 200 μL beads).
• Collect the unlabeled cells in a 15 mL tube.
• Centrifuge at 300 ×*g* for 10 min at 4°C and collect cell pellets for the next steps.

RBC removal:

• Collect the cell pellets and suspend them in 20 times volume of 1 × Red Blood Cell Lysis Solution (∼1 mL per sample).
• After incubating the samples at 4°C for 10 min, add 10 mL of 0.5% BSA buffer and centrifuge them at 300 ×*g* for 5 min. Cell pellets proceed to the FACS step.

NOTE:

(a) The volume of Myelin Removal Beads used depends on the tissue weight. If the mouse brain is greater than 500 mg, more beads may be needed to remove myelin debris.
(b) The LS column should be rinsed with 3 mL of buffer before the suspended cells are applied to it.
(c) Carefully avoid air bubbles when eluting cells, as they may block columns.

### Tissue Dissociation With Percoll

The manual Percoll dissociation procedure was performed based on a published protocol (Lelios and Greter, 2014). Briefly, fresh brain tissue was collected, cut, and digested in 0.4 mg/mL collagenase IV (Sigma-Aldrich) for 45 min at 37°C. Tissue was homogenized by passing it through a syringe with an 18G, 1½ inch needle. The cell suspension was then passed through a 70-micron cell strainer. Cells were washed in PBS, mixed with Percoll (Cat #17089102, GE Healthcare Life Sciences), and then centrifuged at 15,000 × *g* for 30 min. After the topmost layer was discarded, the remainder was filtered through a 70-micron cell strainer. Finally, the cell suspension was centrifuged at 450 ×*g* for 5 min at 4°C and the cell pellet collected for the FACS step.

#### FACS

Cell surface staining: 50–60 min

• Incubate the cells in blocking buffer for 10 min at 4°C.
• Incubate cells with the primary antibodies for 30 min at 4°C.
  - To observe cell morphology and measure pro-inflammatory cytokines and mRNA, we used FITC-CD11b (1:10), PE-CD45 (1:10), and APC-Ly6g (1:10).
  - For evaluation of cell phagocytic function, we used FITC-CD11b (1:10) and BV421-CD45 (1:10).
• Use the corresponding isotype antibodies as negative controls (Supplementary Figure 1).
• Stained samples are washed three times and centrifuged at 300 ×*g* for 5 min at 4°C. Resuspend cells in 0.5–1 mL filtered flow buffer. Add PI to distinguish dead cells.

NOTE:

(a) Cells should be kept on ice until being sorted on a MoFlo cytometer (Beckman Coulter).
(b) The volume of blocking buffer depends on the total cell number counted before staining. We recommend 1 × 10^6^ cells/200 μL buffer for blocking.
(c) Antibodies can be added directly into blocking buffer at the ratio we suggested.
(d) Alternatives:
  For *ex vivo* cell culture, cells can be sorted into culture medium.
  For mRNA extraction and real-time PCR, cells can be sorted into TRIzol reagent.
(e) Cell sorting time depends on the live cell numbers; it usually takes 20–30 min to run each mouse sample.

### Other Methods

#### mRNA Extraction and Real-Time PCR

TRIzol reagent was added to the pellets, which were triturated and incubated for 5 min at room temperature. Then, after 100 μL of chloroform was added to the lysate (200 μL/1 mL TRIzol), the samples were incubated for 2 min and centrifuged at 12,000 ×*g* for 15 min at 4°C. We transferred the aqueous phase, which contained the RNA, to a new RNase/DNase-free tube and added 5 μg glycogen as a carrier and 250 μL isopropanol to each sample. The samples were incubated for 10 min and then centrifuged at 12,000 ×*g* for 10 min at 4°C. We then discarded the supernatants, washed the pellets with 500 μL of 75% ethanol (1 mL/1 mL TRIzol), and centrifuged them again at 7,500 ×*g* for 5 min at 4°C. Supernatants were discarded and the RNA pellets air dried. We used 10–15 μL warm (55°C) RNase/DNase-free water to dissolve the pellets. The RNA concentration was measured on a NanoDrop 2000 spectrophotometer.

For the real-time PCR assay, we followed the protocol provided by the manufacturer. Briefly, for the reverse transcription (RT) reaction, reaction mix (4 μL), enzyme mix (2 μL), RNA (500 ng), and DEPC-treated water were mixed to a total volume of 20 μL. We used the RT reaction program provided by the manufacturer. To measure mRNA, we prepared the reaction mix by combining 7.5 μL TaqMan Universal Master Mix II (2×), 1 μL primers, 50 ng cDNA template, and RNase/DNase-free water to a total volume of 15 μL. Mixtures were transferred to MicroAmp Fast Optical 96-Well Reaction Plates (cat #4346907) for use in the Applied Biosystems 7500 Fast Real-time PCR system (Thermo Fisher Scientific) (Li et al., 2017c). Reactions were incubated at 50°C for 2 min, 95°C for 10 min, and for 40 cycles of 95°C for 15 s and 60°C for 1 min. Relative mRNA expression was calculated by 2^-ΔΔCt^, and GAPDH was used as an internal control. The following TaqMan Gene Expression Assay Mixes (Applied Biosystems) were used: *IL-1β* (Mm00434228_m1), *CD32* (Mm004388875_m1), *IL-10* (Mm00439614_m1), *YM-1* (Mm00657889_m1), *Teme119* (Mm00525305_m1), *Sall1* (Mn00491266_m1), *Ccr2* (Mm00438270_m1), *CD69* (Mm01183378_m1), and *GAPDH* (Mm99999915_g1).

#### Microglial and Macrophage Culture

CD11b^+^/CD45^Int^ (microglia) and CD11b^+^/CD45^high^ (monocytes/macrophages) were sorted in DMEM/F-12 with FBS and penicillin-streptomycin at 4°C. Cell supernatants were centrifuged at 300 ×*g* for 10 min and resuspended in culture medium. Cells were seeded at 5,000 per well on poly-l-lysine-coated Nunc Lab-Tek II Chamber Slides (Thermo Fisher Scientific, cat #154534) and cultured at 37°C in 5% CO_2_. The medium was changed after 24 h. To detect the specific gene expression in cultured microglia and macrophages, we photographed cell morphology under a microscope and then collected cells for mRNA measurement on day 3. At the same time point, lipopolysaccharide (LPS; 25 ng/mL, Sigma-Aldrich, cat #L2630) was added to another set of microglia or monocytes/macrophages for 12 h (Lan et al., 2011). Cell supernatants were collected for ELISA (R&D Systems, Minneapolis, MN), and cells were fixed in 4% paraformaldehyde for immunofluorescence microscopy (Nikon Eclipse 90i fluorescence microscope) (Lan et al., 2017b).

#### Phagocytosis Assay

pHrodo Red Zymosan Bioparticles Conjugates were dissolved in DMEM/F-12 at 100 μg/mL. Then each well of cells was incubated with 100 μL of the beads for 4–6 h at 37°C. Cells were then washed three times in PBS and fixed in 4% paraformaldehyde for immunofluorescence microscopy.

### Statistics

Data are presented as bar graphs (mean ± SD) or dot plots. We made two-group comparisons with a two-tailed Student’s *t*-test followed by Welch’s correction. One-way ANOVA was used for comparisons among multiple groups; Dunn’s *post hoc* analysis was used to determine where those differences occurred. All analyses were carried out with GraphPad Software (GraphPad Prism 5.0; GraphPad Software, Inc., La Jolla, CA). The criterion for statistical significance was *p* < 0.05.

## Results

### Cell Counts After Brain Dissociation

After tissue dissociation and removal of myelin debris and red blood cells, we counted live cells in each group at the 24-h time point. Using our method (Table 1), we collected approximately 2.13 ± 0.23 × 10^6^ cells from each sham mouse (*n* = 6), 1.51 ± 0.22 × 10^6^ cells from each mouse in the blood-induced ICH group (*n* = 6), and 1.67 ± 0.12 × 10^6^ cells from each mouse in the collagenase-induced ICH group (*n* = 6). Using the Percoll protocol (Table 1), we collected 1.92 ± 0.41 × 10^6^ cells from each sham mouse (*n* = 3) and 1.32 ± 0.33 × 10^6^ cells from each mouse in the collagenase-induced ICH group (*n* = 3).

**Table 1:** Cell numbers by gentleMACS and Percoll methods.

	GentleMACS with Myelin Removal Beads (*n* = 6)		Tissue dissociation with Percoll (*n* = 3)	
	Sham	ICH day 1	Sham	ICH day 1
Cell counts before FACS	(2.13 ± 0.23) × 10^6^	(1.67 ± 0.12) × 10^6^	(1.92 ± 0.41) × 10^6^	(1.32 ± 0.33) × 10^6^
Total events collected by FACS (per mouse)	(1.56 ± 0.19) × 10^6^	(1.02 ± 0.22) × 10^6^	(1.01 ± 0.21) × 10^6^	(0.6 ± 0.11) × 10^6^
(FSC-H+SSC-H) subtype (% of total events)	27.9%	22.5%	31.2%	25.8%
No. of microglia	(13.2 ± 2.13) × 10^3^	(16.4 ± 3.52) × 10^3^	(12.8 ± 2.11) × 10^3^	(12.1 ± 3.33) × 10^3^
No. of monocytes/macrophages	(2.12 ± 0.52) × 10^3^	(6.8 ± 1.52) × 10^3^	(2.91 ± 0.24) × 10^3^	(4.13 ± 0.92) × 10^3^

*ICH, intracerebral hemorrhage; FACS, fluorescent-activated cell sorting; FSC-H, forward scatter height; SSC-H, side scatter height.*

### Flow Cytometry and FACS

**FIGURE 3:**
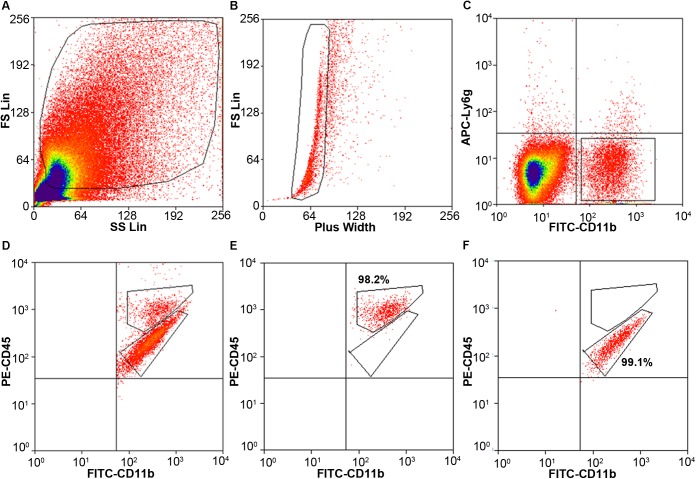
Cell gating by flow cytometry. Eight- to ten-week-old male C57BL/6 mice underwent collagenase injection. Mice were sacrificed on day 1 post-ICH. Mouse brain was perfused with phosphate-buffered saline and dissociated into single cells. Cells were stained with APC-Ly6g, FITC-CD11b, PE-CD45, and propidium iodide (PI). **(A)** Representative gating of all cells. **(B)** Representative gating of singlets. **(C)** Representative gating of Ly6g^-^CD11b^+^ PI^-^ cells. **(D)** Representative gating of CD11b^+^CD45^high^ and CD11b^+^CD45^Int^ after gating from **(C)**. **(E,F)** the post-sort purity of monocytes/macrophages **(E)** and microglia **(F)**.

**FIGURE 4:**
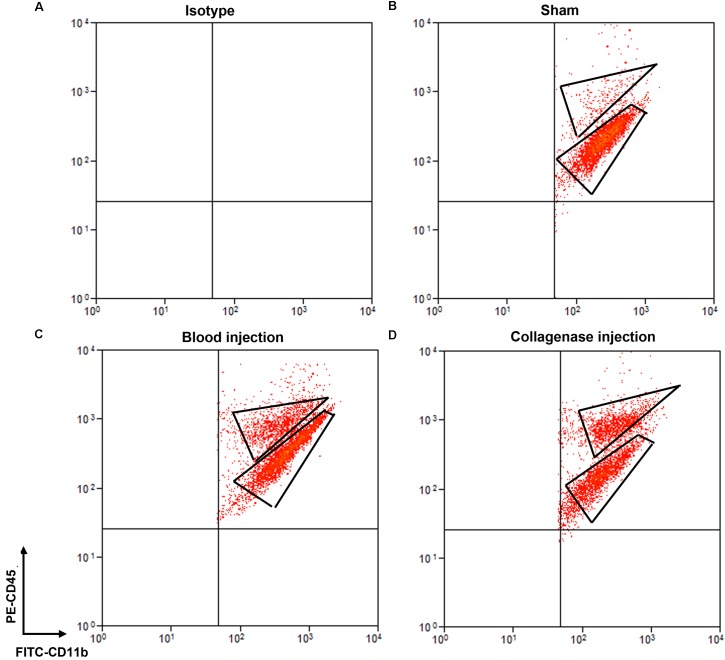
Separation of microglia and monocytes/macrophages by flow cytometry on day 1 after blood-induced or collagenase-induced ICH. Eight- to ten-week-old male C57BL/6 mice underwent collagenase injection, blood injection, or sham procedure. Mice were sacrificed at day 1 post-ICH. Mouse brain was perfused with phosphate-buffered saline and dissociated into single cells. Cells were stained with propidium iodide (PI) and isotype **(A)** or APC-Ly6g, FITC-CD11b, and PE-CD45 **(B–D)** for flow cytometry. Representative images show cell populations in each group, and gates show cells sorted into Ly6g^-^CD11b^+^CD45^high^PI^-^ and Ly6g^-^CD11b^+^CD45^Int^PI^-^ populations.

Cells stained for cell surface markers and with PI were analyzed by flow cytometry. We gated Ly6g^-^CD11b^+^PI^-^ cells (Figure 3) and sorted the two populations of microglia and monocytes/macrophages into the culture medium. The post-sort purity was 98.2% for monocyte/macrophages (Figure 3E) and 99.1% for microglia (Figure 3F). APC-Ly6g^-^FITC-CD11b^+^BV421-CD45^Int^PI^-^ and APC-Ly6g^-^FITC-CD11b^+^BV421-CD45^high^PI^-^ cell populations were sorted for cell phagocytosis measurement; APC-Ly6g^-^FITC-CD11b^+^PE-CD45^Int^PI^-^ and APC-Ly6g^-^FITC-CD11b^+^PE-CD45^high^PI^-^ cell populations were sorted for other experiments. Our results showed that the Ly6g^-^CD11b^+^CD45^Int^ population, which comprises microglia, and the Ly6g^-^CD11b^+^CD45^high^ population, which comprises infiltrating monocytes/macrophages (Greter et al., 2015; Varvel et al., 2016), were clearly separated by CD11b and CD45 staining at 24 h post-ICH in both mouse models (Figure 4). Limited blood-brain barrier damage caused by needle insertion accounts for the small number of CD11b^+^CD45^high^ cells in the sham group (Figure 4B). No positive staining was observed in the isotype group (Figure 4A). Cells were then used for *in vitro* culture and mRNA measurement.

We also compared our method with the classic Percoll method. With our method (Table 1), 1.02 × 10^6^ events were counted by flow cytometry for each collagenase-induced ICH mouse brain, and the (FSC height + SSC height) subtype, gated as live cells, was 22.5% of total counts (Figure 3). With the Percoll method (Table 1), the total number of events for each collagenase-induced ICH mouse brain was 0.6 × 10^6^, and the (FSC height + SSC height) subtype was 25.8% (Supplementary Figure 2). After gentleMACS dissociation and cell sorting, 6.8 ± 1.52 × 10^3^ Ly6g^-^CD11b^+^CD45^high^PI^-^ monocytes/macrophages and 16.4 ± 3.52 × 10^3^ Ly6g^-^CD11b^+^CD45^Int^PI^-^ microglia were collected, respectively, from collagenase-induced ICH mouse brain (*n* = 6). By the Percoll method, 4.13 ± 0.92 × 10^3^ monocytes/macrophages and 12.1 ± 3.33 × 10^3^ microglia were sorted from the ICH mice (*n* = 3).

### Purity of Microglia and Monocyte-Derived Macrophages in Sorted and Cultured Cells

**FIGURE 5:**
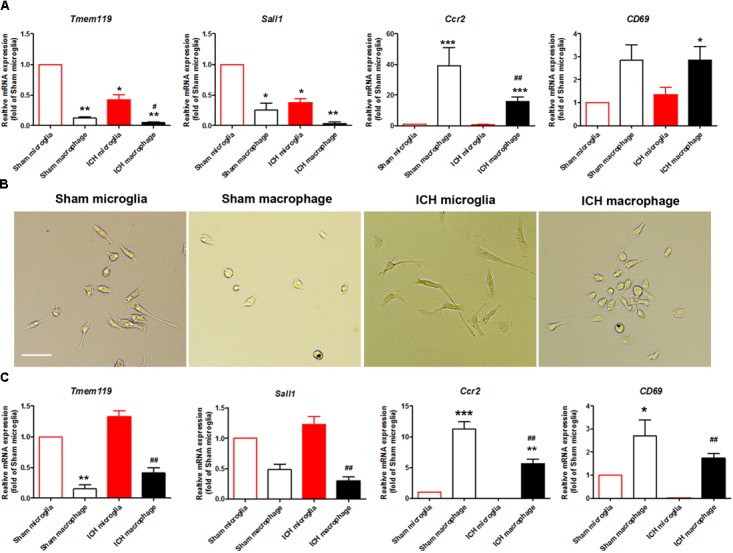
Expression of microglia- and monocyte/macrophage-specific markers in sorted and cultured cells after ICH. Eight- to ten-week-old male C57BL/6 mice underwent collagenase injection or sham procedure. Mice were sacrificed at day 1 post-ICH. Mouse brain was perfused with phosphate-buffered saline and dissociated into single cells. Microglia (Ly6g^-^CD11b^+^CD45^Int^PI^-^) and monocytes/macrophages (Ly6g^-^CD11b^+^CD45^high^PI^-^) were isolated and sorted. **(A)** mRNA from cells was extracted and real-time PCR carried out with different primers. *GAPDH* was used as an internal control, and results are shown as fold of control. **(B)** Sorted cells were seeded and bright field images were taken after 3 days in culture. Representative images are shown. **(C)** mRNA was extracted after sorted cells were cultured for 3 days. Real-time PCR was carried out with different primers. *GAPDH* was used as an internal control, and results are shown as fold of sham microglia. ^∗^*p* < 0.05, ^∗∗^*p* < 0.01, ^∗∗∗^*p* < 0.001 vs. corresponding control; #*p* < 0.05, ##*p* < 0.01 vs. corresponding group in sham animals; *n* = 3. Results are presented as mean ± SD. One-way ANOVA followed by Dunn’s multiple comparison post-test. Scale bars: **(B)** 20 μm.

The literature has reported that Tmem119 and Sall1 are microglia-specific markers (Bennett et al., 2016; Buttgereit et al., 2016), that Ccr2 is a monocyte/macrophage-specific marker (Gu et al., 2016), and that CD69 is enriched in monocytes/macrophages but does not change in activated microglia (Lewis et al., 2014). To confirm the purity of the two sorted populations, we extracted mRNA and performed real-time PCR with sorted Ly6g^-^CD11b^+^CD45^Int^ and Ly6g^-^CD11b^+^CD45^high^ cells. As expected, *Tmem119* and *Sall1* were more highly expressed in the microglial cell population, whereas *Ccr2* and *CD69* were more highly expressed in the monocyte/macrophage cell population (Figure 5A). The expression levels of *Tmem119*, *Sall1*, and *Ccr2* were decreased at 1-day post-ICH compared with those in sham animals (Figure 5A). After the sorted cells had been cultured for 3 days, we observed morphologic differences between microglia and macrophages, and microglia from the ICH group exhibited morphology typical of activated microglia (Figure 5B). Subsequently, we performed real-time RT PCR and verified the specific markers of these two cell populations. As expected, *Tmem119* and *Sall1* were still expressed mainly in microglia, and *Ccr2* and *CD69* were expressed mainly in monocytes/macrophages (Figure 5C). However, the expression level of *Tmem119* and *Sall1* was higher in the ICH group than in the sham group, whereas the expression levels of *Ccr2* and *CD69* remained lower in the ICH group than in the sham group (Figure 5C). These results indicate that the expression of those genes is differentially regulated by ICH and by *in vitro* culturing.

### LPS Response and Phagocytosis by Sorted Microglia and Macrophages *in vitro*

**FIGURE 6:**
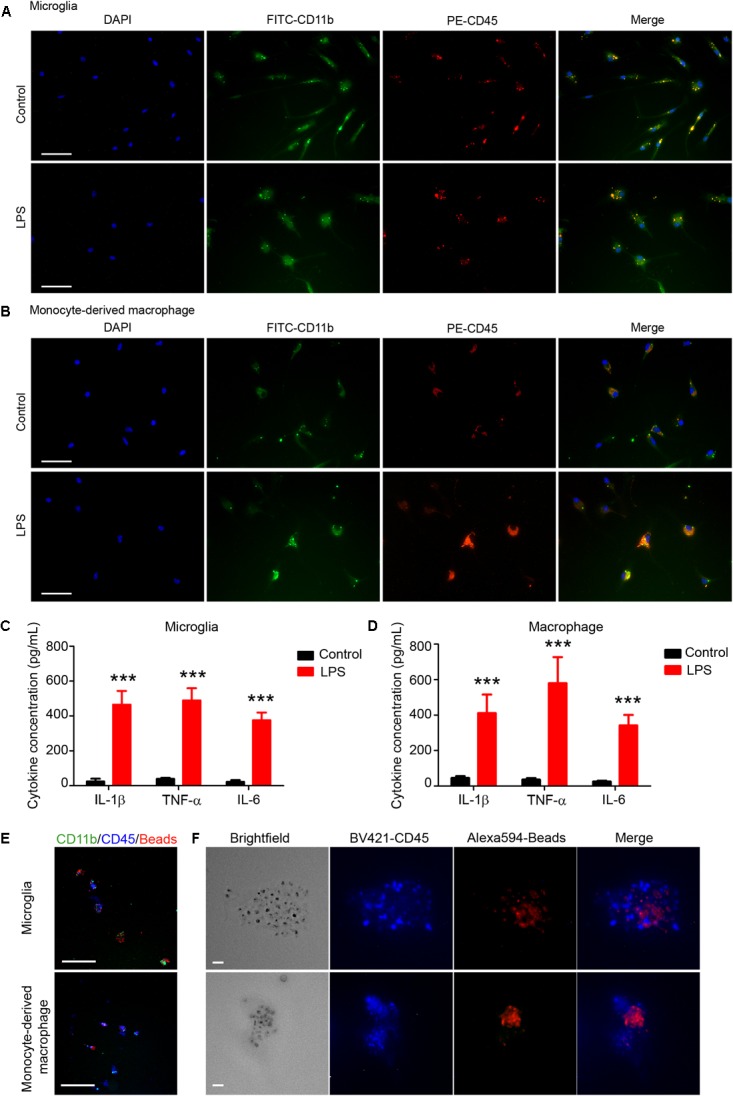
Sorted microglia and monocytes/macrophages respond to lipopolysaccharide (LPS) stimulation and phagocytose beads *ex vivo*. At 1 day after mice were injected with collagenase, microglia (Ly6g^-^CD11b^+^CD45^Int^PI^-^) and macrophages (Ly6g^-^CD11b^+^CD45^high^PI^-^) were isolated, sorted, and seeded onto slides. Cells recovered briefly and were incubated with LPS or vehicle for 12 h. Representative images of **(A)** microglia and **(B)** monocyte-derived macrophages are shown. **(C,D)** Conditioned medium was collected and ELISA assays were performed. ^∗∗∗^*p* < 0.001 vs. corresponding control; *n* = 3. Results are presented as mean ± SD. Two tailed Student’s *t*-test followed by Welch’s correction. **(E,F)** Cells were incubated with fluorescence-conjugated latex beads for 4–6 h and fixed before imaging. Representative images are shown. Scale bars: **(A,B)** 50 μm; **(E)** 100 μm; and **(F)** 10 μm.

Microglia and monocyte-derived macrophages showed healthy morphology with FITC-CD11b, PE-CD45, and DAPI staining (Figures 6A,B, first row). After LPS stimulation (Figures 6A,B, second row), microglia (Figure 6A) and monocytes/macrophages (Figure 6B) exhibited reactivated morphologies, with larger cell bodies and more dendrite branches than the control group.

ELISA assays showed that levels of proinflammatory cytokines (IL-1β, TNF-α, and IL-6) were significantly higher in the LPS-induced microglia (Figure 6C) and macrophages (Figure 6D) than in the control groups. Furthermore, we used APC-Ly6g, FITC-CD11b, and BV421-CD45 to sort microglia and monocytes/macrophages in a parallel experiment. We found that sorted cells were able to phagocytose fluorescent latex beads *in vitro* (Figures 6E,F). These results indicate that the sorted microglia and monocytes/macrophages maintained their inflammatory reactivity to LPS and their phagocytic function.

### M1 and M2 Marker mRNA Expression in Microglia and Monocytes/Macrophages

**FIGURE 7:**
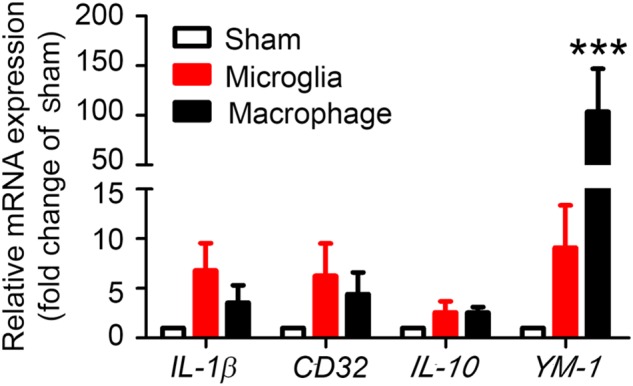
Sorted microglia and monocytes/macrophages express M1 and M2 markers *ex vivo*. At 1 day after mice underwent collagenase injection or sham procedure, microglia (Ly6g^-^CD11b^+^CD45^Int^PI^-^) and monocytes/macrophages (Ly6g^-^CD11b^+^CD45^high^PI^-^) were isolated and sorted. mRNA from cells was extracted and real-time PCR carried out with different primers. *GAPDH* was used as an internal control, and results are shown as fold of sham. ^∗∗∗^*p* < 0.001 vs. corresponding control; *n* = 3. Results are presented as mean ± SD. One-way ANOVA followed by Dunn’s multiple comparison post-test.

To evaluate whether our method can be used to observe MMϕ polarization after ICH, we extracted mRNA and performed real-time PCR with sorted Ly6g^-^CD11b^+^CD45^Int^PI^-^ and Ly6g^-^CD11b^+^CD45^high^PI^-^ cells. Our results showed that M1 markers, *IL-1β* and *CD32*, and M2 markers, *IL-10* and *YM-1*, were increased in both microglia and monocytes/macrophages (Figure 7). Notably, *YM-1* was significantly elevated in the monocyte/macrophage population on day 1 post-ICH (Figure 7).

### Potential Pitfalls and Troubleshooting

#### Isolate Microglia/Macrophages at Different Time Points Post-ICH and From Sham-Operated Group

The number of infiltrating MMϕ differs at different time points after ICH. Therefore, sorted MMϕ may not be sufficient for mRNA extraction or cell culture. To isolate MMϕ more than 5 days after ICH, we recommend pooling two or more mouse brains together to collect enough cells. To collect enough macrophages from sham-operated mice, we suggest pooling at least three mouse brains together.

#### Maintain Cell Viability Before/During FACS

Brain cells are more vulnerable than other cells (tumor cells or blood cells), and hemorrhagic brain contains more debris than non-traumatic brain tissue. Thus, tissues/cells need to be kept on ice at all times except during enzyme incubation. All procedures require quick (perfusion) and gentle (incubation and resuspension) techniques. If the FACSorting takes more than 3 h, we suggest that cells be resuspended in Neurobasal medium (cat. 21103049, Thermo Fisher Scientific) with 1% BSA to maximally maintain cell viability.

#### Cell Collection After FACS

In some cases, the number of sorted cells may be small. To maximally preserve the viability and number of sorted cells, we recommend seeding cells directly onto poly-l-lysine–coated cover slips or 6- or 12-well plates. Cells can be sorted directly into TRIzol reagent and stored at –80°C for up to 6 months before mRNA is extracted.

## Discussion

Microglia and monocyte-derived macrophage activation and polarization play an important role in hematoma resolution after ICH (Mracsko and Veltkamp, 2014; Zhou et al., 2014; Lan et al., 2017c; Zhang et al., 2017). In preclinical studies, upregulation of M2-like MMϕ has been shown to ameliorate outcomes after ICH (Wan et al., 2016; Yang J. et al., 2016; Chang et al., 2017; Pascual et al., 2017; Yu et al., 2017; Zhou et al., 2017). However, the concept of MMϕ phenotypes in these studies was unclear. Most evaluations of MMϕ polarization use whole brain homogenates in Western blotting, ELISA, or real-time PCR to detect changes in protein or mRNA expression. However, owing to the global expression of most proinflammatory and anti-inflammatory cell markers on neurons, astrocytes, MMϕ, and even oligodendrocytes (Ren and Dubner, 2008), these methods cannot be used to precisely determine the dynamic changes in MMϕ after ICH. Furthermore, recent studies indicated that the roles of microglia and monocyte-derived macrophages might differ after ICH: the infiltrating CCR2^+^Ly6c^high^ cells accelerated neurologic deficits in the acute phase after ICH (Hammond et al., 2014), and the activity of CD11b^+^/CD45^high^ cells was similar to that of monocytes, which contribute to brain injury post-ICH (Min et al., 2016). However, microglial depletion was beneficial for brain recovery on day 3 post-ICH (Li et al., 2017a). These results indicate that evaluating the functions of microglia and monocyte-derived macrophages separately is critical in preclinical and clinical studies of ICH (Lan et al., 2018). Therefore, a protocol is needed that can reliably sort microglia and monocyte-derived macrophages from the ICH brain.

Several methods have been developed to separate microglia and monocytes/macrophages in the mouse ICH brain. The traditional method for distinguishing resident microglia from infiltrating monocytes/macrophages is based on cell morphology (amoeboid, ramified or reactivated (Jeong et al., 2013; Li et al., 2018)) and CD45 staining (Perego et al., 2011). However, this method can only identify cellular location (in the hematoma core or perihematoma regions) and marker expression (by immunostaining other cell markers); the ability to assess cellular function is very limited. Another method is to generate bone marrow chimeras using Cx3cr1^gfp/+^ transgenic (Ajami et al., 2007; Tashima et al., 2016) or other transgenic mice. This approach allows researchers to separate the infiltrating bone marrow-derived monocytes/macrophages (Cx3cr1^gfp/+^) from resident microglia (Cx3cr1^-/-^). Unfortunately, bone marrow transplantation has the potential to cause acute and chronic inflammatory responses (Mildner et al., 2007), which may affect the MMϕ function and even alter the expression of some cell surface markers (Moravan et al., 2016). Recently, researchers developed new tools based on cell surface markers to distinguish microglia and infiltrating monocytes/macrophages. The CX3CR1^CreER/+^:R26^IDTR/+^ transgenic mouse was developed to enable microglia and monocytes/macrophages to be observed separately (Parkhurst et al., 2013). Moreover, a new anti-TMEM119 antibody, which can specifically identify microglia in the brain, was applied to study microglia and monocyte-derived macrophage differences (Bennett et al., 2016). Nevertheless, even as new approaches and tools are developed to study MMϕ, flow cytometry-based FACS remains the gold standard to separate microglia and monocytes/macrophages for *ex vivo* and *in vitro* ICH study, as this method allows maximal retention of cell properties after brain damage.

In the present study, we used Ly6g, CD11b, and CD45 antibodies to label microglia and monocytes/macrophages, as these are classical cell markers of these two populations. As an alternative, CD44 is also a good marker to distinguish microglia, monocytes, and monocyte-derived macrophages after brain injury. Studies showed that the CD11b^high^CD44^low^ population was also CD45^Int^ and Ly6c^low^, and that the CD11b^high^CD44^high^ population was CD45^high^ and Ly6c^high^ in an experimental autoimmune encephalomyelitis (EAE) model (Lewis et al., 2014). Therefore, the combination of cell markers CD44, CD11b, CD45, and Ly6g could potentially be more reliable for separating microglia, monocytes, and macrophages. It will be valuable in the future to perform another series of experiments to show that this combination of markers is also useful after ICH.

The tissue dissociation efficiency is a key factor that affects the activity of sorted cells. In our optimized protocol, we chose the gentleMACS dissociation system, which is flexible and fast and allows the preparation of eight samples simultaneously. To isolate live cells from adult mouse brain, we used Myelin Removal Beads to decrease the debris content and maintain cell viability. Furthermore, because of the substantial bleeding in a hemorrhagic brain, we also added double volume of Red Blood Cell Lysis Solution to maximally eliminate the effect of autofluorescence. The dissociation process can be completed in approximately 2 h and is not affected by the sample number (up to 8); the whole procedure, including sorting, can be finished in 8 h. More importantly, the cell viability and status are not heavily dependent on the proficiency of the operator. Thus, variability between groups within and between experiments, even those carried out by different individuals, is minimal, making data reliable and reproducible. Additionally, the process produces approximately 1.5–3.0 × 10^6^ live cells that can be collected for subsequent FACS. This number should be sufficient for follow-up experiments (e.g., immunostaining, real-time PCR, and nanostring) without needing to pool several brains together in most cases, which could potentially increase variability. Compared to the classic Percoll-based dissociation protocol, we found no significant differences in the percentage of FSC height + SSC height subtype or microglia/macrophage cell numbers by FACS. However, the total number of events collected for each sample was less with the Percoll method than with our method on day 1 post-ICH.

After flow cytometry, two populations of CD11b^+^CD45^Int^ (microglia) and CD11b^+^CD45^high^ (monocytes/macrophages) can be clearly separated from mice with ICH. However, we did observe a small macrophage population in sham mice. We also used Ly6g antibody to distinguish neutrophils from monocytes. We gated Ly6g^-^/CD11b^+^ and sorted microglia and monocytes/macrophages separately. One alternative approach is to use Cx3cr1^GFP/+^CCR2^RFP/+^ mice (Mizutani et al., 2012) and gate Cx3cr1^+^CCR2^-^ to separate monocytes from MMϕ. To confirm the purity of the gated cells, we performed real-time RT PCR to examine specific markers: *Tmem119* and *Sall1* for microglia, *Ccr2* and *CD69* for monocyte/macrophages. As expected, *Tmem119* and *Sall1* were expressed mainly in the microglial (CD11b^+^CD45^Int^) population, and *Ccr2* and *CD69* were expressed mainly in the monocyte/macrophage population (CD11b^+^CD45^high^). Importantly, cultured cells retained the specific marker expression for days post-sorting.

We also found that the expression level of those markers changes after acute ICH. *Tmem119* is a recently discovered specific microglial marker in the brain (Bennett et al., 2016). However, its biologic function is unknown. *Tmem119* expression was shown to be elevated in the brains of patients with Alzheimer’s disease, but not in those with amyotrophic lateral sclerosis or Parkinson’s disease (Satoh et al., 2016). Interestingly, *Tmem119* is a target gene of TGFβ1 in postnatal microglia but cannot be regulated by TGFβ1 in mature microglia (Attaai et al., 2018). Our results showed that *Tmem119* expression significantly decreased on day 1 post-ICH, suggesting that it may participate in microglial function after acute brain injury. *Sall1* is another important transcriptional regulator that is required for microglia to retain a healthy phenotype and characteristics (Buttgereit et al., 2016). Similar to *Tmem119*, *Sall1* was significantly suppressed on day 1 post-ICH; however, after 3 days of culture, this reduction disappeared in ICH microglia, perhaps suggesting self-recovery. Therefore, whether the reduction of *Sall1* expression can be used as a biomarker for ICH diagnosis needs further investigation.

It has been reported that in an EAE animal model, Ccr2 was rarely expressed in microglia but highly expressed in monocyte-derived macrophages. Moreover, Ccr2 expression decreased in macrophages of mice with EAE scores of 0 and 3 compared with that of naïve mice (Lewis et al., 2014). Our data indicated that Ccr2 is a specific monocyte/monocyte-derived macrophage marker after ICH, and its expression was downregulated after ICH. Unlike Ccr2, CD69 was highly expressed in macrophages, but we did not observe a significant change in *CD69* expression on day 1 post-ICH. Therefore, a time-course experiment is needed to study the dynamic expression changes and function of CD69 after ICH.

For *in vitro* studies, researchers commonly use primary microglia from p0 mouse pups to investigate microglial function. However, these cells are not completely mature (Moussaud and Draheim, 2010). Moreover, astrocyte-contaminated microglia may have altered cytokine production after stimulation (Bohlen et al., 2017). With our protocol, the sorted cells are mature, can be cultured *in vitro*, retain a healthy morphology, and can be reactivated by stimulation with LPS for proinflammatory cytokine upregulation. Furthermore, the microglia we collected maintained the ability to engulf beads by phagocytosis. These results provide evidence that the sorted MMϕ have excellent viability, activity, and function. When we measured levels of extracted mRNA by real-time PCR, we were able to examine the changes in microglial activation and polarization more precisely, accurately, and efficiently than is possible with immunostaining. In a future study, we will apply nanostring technology and proteomics to further investigate the changes in microglia and monocyte-derived macrophages after ICH.

This protocol has several limitations. First, the MACS sorter, dissociation kit, and C tubes are relatively more expensive than components needed for the traditional method. Second, to achieve the best results, different dissociation kits need to be used for neonatal and adult mice. In addition, because massive bleeding is induced in the ICH model, it is difficult to completely remove red blood cells. Third, gating of the monocyte-derived macrophages would be different from gating of the monocytes because transformation of monocytes into macrophages alters cell size and changes surface marker expression in the ICH animals at different time points. Thus, to investigate the transformation of monocytes into macrophages after ICH, we will sort Ly6c^+^Ly6g^-^CD11b^+^CD45^high^ monocytes and Ly6c^-/-and+/-^Ly6g^-^CD11b^+^CD45^high^ macrophages separately in our future research.

## Conclusion

In conclusion, we established a method based on MACS dissociation and sorting by FACS flow cytometry to separate microglia and monocyte-derived macrophages after ICH. The whole process can be achieved within 5–8 h, and the sorted microglia and monocyte-derived macrophages maintain their post-ICH characteristics, an attribute that is critical for ICH research. This fast, efficient, and precise protocol will be valuable for multiple applications and an important means by which to study the role of microglia and monocyte-derived macrophages after ICH and other brain diseases.

## Author Contributions

QL, XL, and JW designed the experiments and wrote the manuscript. QL, XL, XH, and JW analyzed the data.

## Conflict of Interest Statement

The authors declare that the research was conducted in the absence of any commercial or financial relationships that could be construed as a potential conflict of interest.

## Abbreviations

FACS: fluorescence-activated cell sorting
ICH: intracerebral hemorrhage
MACS: magnetic-activated cell separation
MMϕ: microglia/macrophages
PI: propidium iodide
