# Supplementary material for: Expression of Tmem119/Sall1 and Ccr2/CD69 in FACS-Sorted Microglia- and Monocyte/Macrophage-Enriched Cell Populations After Intracerebral Hemorrhage
Source: Front Cell Neurosci. 2019 Jan 9;12:520. doi: 10.3389/fncel.2018.00520 (PMC6333739; doi:10.3389/fncel.2018.00520)
Supplement: Supplementary file 1 [file Data_Sheet_1.pdf]

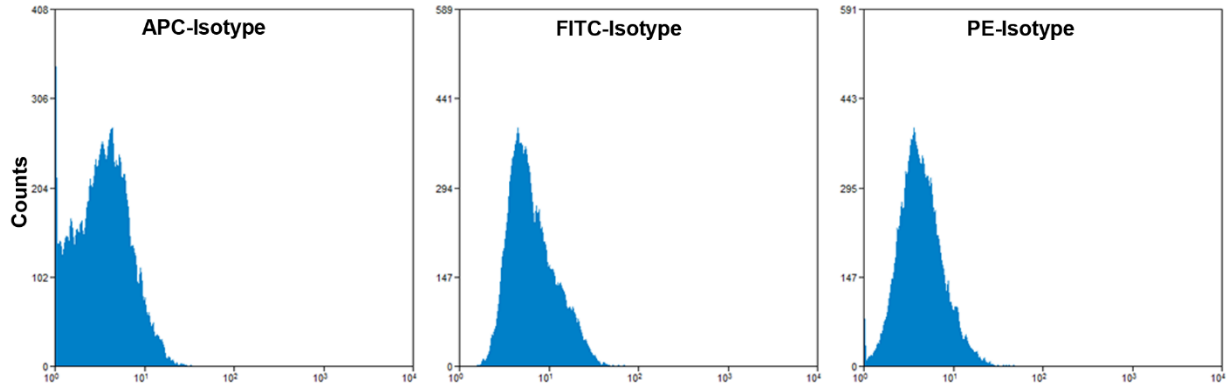

Supplementary Fig. 1 Isotype histogram images. Isotypes of each antibody (APC-Ly6g, FITC-CD11b, and PE-CD45) were used as controls.

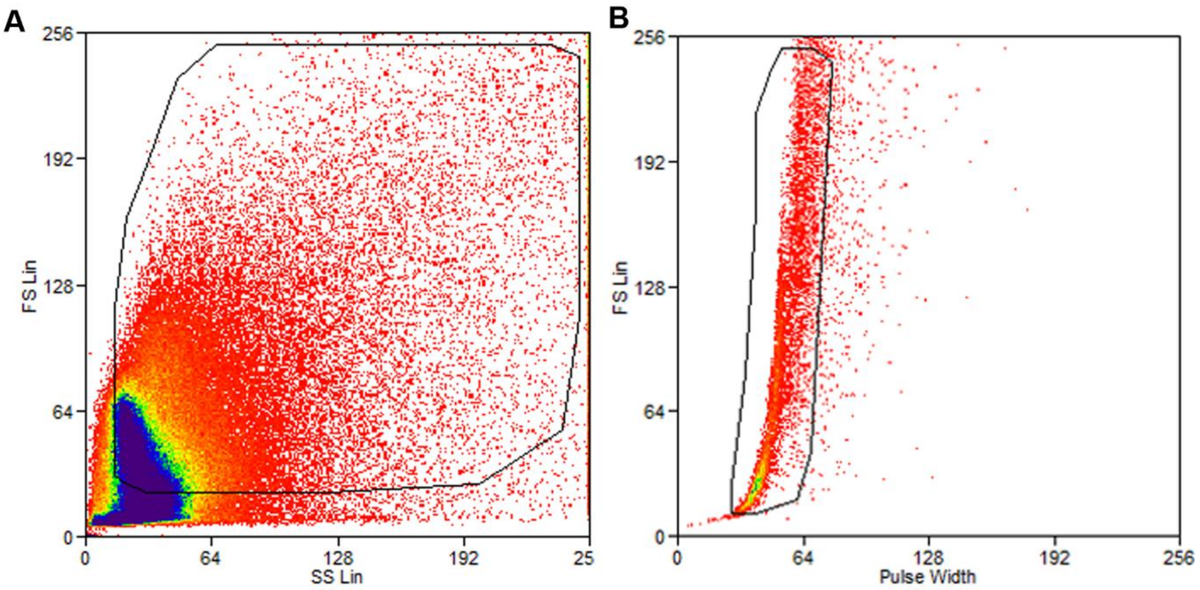

Supplementary Fig. 2 Flow cytometry by Percoll method. Eight- to ten-week-old male C57BL/6 mice underwent collagenase injection. Mice were sacrificed on day 1 post-ICH. Mouse brain was perfused with phosphate-buffered saline and dissociated into single cells. Cells were stained with APC-Ly6g, FITC-CD11b, PE-CD45, and PI. (**A**) Gating of all cells. (**B**) Gating of singlets.
